# Round the Hospitals

**Published:** 1920-09-25

**Authors:** 


### ROUND THE HOSPITALS.

The first autumnal session of the General Nursing Council approaches, and rumour has it that before long now the preliminaries will be settled and the doors of the Registration Office will be opened. It is not too soon to be looking through papers and getting together the necessary credentials. Suppose, for instance, original certificates have been burnt, stolen or lost, steps should be taken at once to appeal to the hospital which granted them for an attested copy, which can usually be had on payment of a small fee. It is well, too, to post up all the details of the nursing career and make sure that all is in order. Nurses who have had a varied career are often puzzled to account for their entire nursing life off-hand. It will probably be a great help in filling in forms of application for State registration if this has been done systematically beforehand, with every date verified.

Nurses attached to the Poor-law service are being besieged for their patronage by four organisations, each of which is entreating them to remember "Short's your friend, not Codlin." The names of these associations are as follows:—National Poor-law Officers' Association; Poor-law Workers' Trade Union of England and Wales; National Union of Trained Nurses; Professional Union of Trained Nurses. Letters have been addressed to various boards of guardians by representatives of all these associations, desirous of being "recognised" or of addressing the nurses. The Poor-law Officers' Association is certainly, we understand, the earliest, and probably stands first in members, for at a time when it was believed that the Provisional Council of the College would be made up of representatives of various nursing interests this body was able to present a petition signed by 4,000 nurses in the Poor-law service, praying for representation. A large number of the members of the nurses' section in this association are now also members of the College.

It is apparent from the remarks of "A Consulting Physician" in a lay paper that there is a good deal of misapprehension about the percentage of probationers who fail in getting through their trial month from reasons of health. In good training-schools failures on this account are very few, partly because weaklings are detected in the medical examination prescribed for all candidates and partly because there is nothing in the probationer's duties which makes an undue strain on the health. Except for the early rising, which at first tries some women not accustomed to it, the routine is particularly bracing, and after the first year a definite improvement in health is usually observable. There is no doubt that one of the useful functions of the preliminary school is to tone up the general health of the candidates, and by physical exercises and the practice of a routine approximating in some degree to that of the hospital to prepare the probationer to take easily to her work.

The withdrawal of cheap fares for patients travelling to convalescent homes would mean a very severe charge upon Samaritan Funds, which are heavily taxed to meet all the demands made upon them. Before the Railway Rates Advisory Committee at Lincoln's Inn, Mr. Courtney Buchanan, of the British Hospitals Association, pleaded the cause of the hospitals, and urged that the present coupons for reduced fares obtainable by patients, officers, and staff be not abolished. The London Hospital, the Metropolitan Asylums Board, and others also appealed against the threatened cutting-off of railway concessions, and the case is so strong that we trust the Committee may see fit to make a recommendation in these special cases.

The ambulance work undertaken by the Red Cross and St. John Voluntary Ambulance Service in peace bids fair to rival what was accomplished in the war. At the distribution of badges and annual inspection of the Brighton and Hove Corps of the St. John Ambulance Brigade and the V.A.D. Sussex 53 on September 11, the Brighton Nursing Division paraded in their grey and white uniforms, the Police Division in blue, and the V.A.D. in khaki. Miss Blanche Fairs stated that the number of patients carried by the ambulance service from January to date was 337, with a mileage of over 4,000. In July, sixty, and in August, eighty patients were conveyed. The ambulances are used by the Ministry of Pensions, the Royal Army Service Corps, the Steyning Board of Guardians, the Hove Corporation, and the Sussex County Hospital, as well as by private paying patients, and no charge is made to the very poor.

**ROUND THE HOSPITALS—(*continued*).**

INTERESTING cookery competitions for nurses will take place next week at the Royal Horticultural Hall, Vincent Square, S.W., in connection with the Cookery and Food Exhibition. Section III (invalid cookery), class thirty-four, is open to trained nurses without entrance fee. It will show trays containing each four dishes suitable for invalids. Another section is open to V.A.D.s., and a third, which is of exceptional interest, is for trained nurses and is limited to vegetarian dishes, soups and beverages. The crux of the meatless dish lies in the flavouring, for when meat is forbidden the cook is usually quite helpless, and dooms the patient to a dreary round of utterly flavourless cereals.

---

THE desire to make nursing a more attractive profession is spreading. In Denmark, Norway, Sweden, and Finland pressing difficulties in the way of attracting probationers to the service are being experienced, and a Congress has been taking place at Copenhagen to exchange views on the subject of an eight-hour day and a general increase of salaries. The cost of food and clothing has risen in European countries far beyond the average in Great Britain, and salaries have by no means yet overtaken it. The Danish Nurses' Council comprises over 4,000 certificated nurses. The problem of promoting co-operation between the nursing authorities in the Northern countries is being eagerly debated, for the prospects of nurses depend very largely on the extent to which this can be brought about.

CRAIG HOUSE, Swansea, for the equipment of which as a maternity centre the American Red Cross Society generously gave £2,000, is now ready for patients, and is admirably adapted for its purpose. It contains nine beds, with good quarters for the staff, and is most comfortable, airy, and cheerful. It has been inspected by Miss Halford, secretary of the National League for Maternity and Child Welfare, which is acting on behalf of the American Red Cross, and she expressed herself "more than delighted" with the house, its equipment and general arrangements. It will be a very great boon in Swansea, where overcrowding is rampant.

---

THE appointment of Miss K. E. Luard, R.R.C., to be matron of the South London Hospital for Women and Children, Clapham Common, is exceedingly popular. Miss Luard gave up an interesting appointment when war broke out, and she joined up as a member of Q.A.I.M.N.S.R. She won several distinctions, including a bar to the R.R.C., and her previous experience and high qualifications marked her out for promotion after her long war service.

---

THE nurses and their friends at St. Bartholomew's have done splendidly to raise £100,000 already for the first block of the new Queen Mary's Nurses' Home, for there has never been a "tighter" season for money. They return to the charge with redoubled energy and hope to obtain the second £100,000 before the end of next year to

**ROUND THE HOSPITALS**—(*continued*).

complete the building. The first block is to be started at once, and it is interesting to know that the suggestion made by the authorities that a donation of £350 will suffice to build and name a room has been well taken up.

---

At the meeting of the Bitterne Park Nursing Association, Lady Swaythling, who took the chair as President, announced that they had at last succeeded in obtaining a fully-qualified nurse in Sister Lagareste, but that much of her time was wasted walking about the district owing to the lack of a bicycle. A subscription was taken up there and then, and the nurse will have her bicycle. It is very unusual to find any nursing association which does not accept liability for the nurses' locomotion, but we believe the time is approaching when in hilly districts some kind of power, electric or petrol, will have to be supplied. We know of one district twenty miles in circumference and containing scarcely twenty yards of level ground, which is by no means exceptional in the demands which it makes on the nurse's time and powers of endurance. From the point of view of economy alone, would not petrol cost less than the labour?

---

Is village nursing a career for educated women? The answer which most people acquainted with the facts would be disposed to make is in the negative. Yet the desire to obtain recruits for the county nursing associations does occasionally lead enthusiasts to speak and write of the village midwife's life rather as it ought to be than as it is. In particular, the authorities of the Herefordshire County Nursing Association appear, from the correspondence in the local press, to be going too far in their eulogy of a service which may be "second to none in usefulness" but is certainly far from first in dignity. The village midwife with her one year's training cannot hope to be eligible for registration as a trained nurse, and wherever she goes beyond her limited sphere must find herself at a disadvantage. It is a service eminently fit for women who, from widowhood or other causes, desire to take up nursing after thirty, and enjoy country life. It has been brought to notice recently by an observant county medical officer that the county nursing associations are not getting the kind of women best suited for this branch of work. It will not help, but will certainly hinder for them to aim at attracting young educated women who ought either to take full training or leave nursing alone.

---

A very disagreeable incident is reported from Ireland in the kidnapping of a nurse, Miss Crowe, who was in attendance on her patient at Victoria Bridge, five miles from Newtownstewart, County Tyrone. Miss Crowe, whose home is in Belfast, was nursing Mr. Archibald Anderson, a retired merchant. She had received threatening letters warning her to leave her patient, but pluckily stood her ground until, at 4 a.m. some few days ago, the house was entered and she was carried forcibly away in a motor-car, attired only in her night clothes, for a distance of seven miles. She found herself deposited by the roadside in the dark, a situation of considerable danger. All combatants are accustomed to respect nurses, and it is a very bad symptom that in Ireland to-day the traditional reverence which hangs about this neutral profession has been more than once openly trampled on.

---

The Aberystwith Infirmary is calling loudly for reconstruction and enlargement, and a successful three-days' bazaar has recently been held to raise the necessary £10,000 required, towards which the British Red Cross Society has offered a grant of £3,000. The institution was built in 1885, and contains twenty-five beds. The plan is to increase the ward accommodation to sixty beds, to provide a separate ward for children, an $x$-ray room, massage and orthopædic departments, and increase the number of private wards to six. The spirit which inspires the Cardigan residents, the cottars, and quarry-men is beyond all praise, and the success of the movement is already assured. We wish they could make the number of beds up to 100 and get it recognised officially as a training-school for nurses.

---

Mrs. Keen, the honorary secretary of the North Islington School for Mothers, has made out a table which reveals the deficiencies presented by nursing as a career for girls. In many careers, that of the teacher, for instance, of the clerk, the typist or commercial assistant, there is a complete chain or ladder extending from the elementary school up to their entry into training for the selected occupation. As regards nursing, there are gaps in the ladder, and the aspirant must either fill in some years with another occupation, at the risk of being absorbed by it, or remain idle. The place, in short, which the trade school, training college, or university takes in the scheme of candidates for other occupations is unfilled. We believe this vital matter of bridging the gulf is by far the most important to be tackled in the difficult art of attracting probationers, and we look with some confidence to the College of Nursing to lead the way.

---

Every good wish for success to the Sheffield Bazaar to be held on September 24 and 25 at the Cutlers' Hall. The promoters are the members of the College of Nursing in that city, with Miss Smeeton, R.R.C., matron of the Royal Infirmary and the acting matron of the Royal Hospital, to take the lead. For some time past the need for a nurses' club in Sheffield has been making itself felt, and the proceeds of the bazaar will, it is hoped, go a long way towards realising this purpose. Nurses, it has been discovered during the last four years, are eminently clubbable, and Yorkshire nurses certainly not least.

